# Mesenchymal Stem Cells in Oral and Maxillofacial Surgery: A Systematic Review of Clinical Applications and Regenerative Outcomes

**DOI:** 10.3390/jcm14113623

**Published:** 2025-05-22

**Authors:** Gianna Dipalma, Grazia Marinelli, Irene Palumbo, Mariafrancesca Guglielmo, Lilla Riccaldo, Roberta Morolla, Francesco Inchingolo, Andrea Palermo, Alessio Danilo Inchingolo, Angelo Michele Inchingolo

**Affiliations:** 1 Department of Interdisciplinary Medicine, University of Bari “Aldo Moro”, 70121 Bari, Italy; giannadipalma@tiscali.it (G.D.); graziamarinelli@live.it (G.M.); irenepalu@icloud.com (I.P.); m.guglielmo2@studenti.uniba.it (M.G.); l.riccaldo@studenti.uniba.it (L.R.); roberta.morolla@uniba.it (R.M.); alessiodanilo.inchingolo@uniba.it (A.D.I.); angeloinchingolo@gmail.com (A.M.I.); 2 Department of Experimental Medicine, University of Salento, 73100 Lecce, Italy; andrea.palermo@unisalento.it

**Keywords:** mesenchymal stem cells, bone regeneration, adipose tissue, bone marrow cells, dental pulp, tissue engineering, reconstructive surgical procedures, tissue scaffold, regenerative medicine

## Abstract

**Aim**: This systematic review aims to evaluate the use of mesenchymal stem cells, particularly those derived from bone marrow, adipose tissue, and dental pulp in maxillofacial and oral surgery, focusing on their regenerative potential, clinical applications, and integration with biomaterials. **Introduction**: Mesenchymal stem cells are multipotent stem cells known for their immunomodulatory and regenerative abilities. Their low immunogenicity and differentiation capacity make them ideal for treating craniofacial defects and enhancing soft tissue repair. **Materials and Methods**: The review followed PRISMA guidelines and was registered in PROSPERO. The literature was searched across PubMed, Scopus, and Web of Science from 2009 to 2024. Twelve studies met the inclusion criteria and were analyzed for clinical efficacy and methodological quality. **Results**: Clinical trials demonstrated the safety and regenerative benefits of mesenchymal stem cell in bone and soft tissue reconstruction. Adipose-derived stem cell and dental pulp stem cell showed favorable outcomes in angiogenesis and healing, while bone marrow’s cell proved effective in bone regeneration, particularly when combined with scaffolds. **Discussion and Conclusions**: Although results are promising, limitations remain in consistency and long-term outcomes. Optimizing scaffold integration, preservation methods, and delivery techniques is crucial. Mesenchymal stem cell-based therapies represent a powerful, minimally invasive alternative to traditional grafting in oral and maxillofacial surgery.

## 1. Introduction

In recent years, regenerative medicine has emerged as a promising frontier in the treatment of various tissue defects and degenerative conditions. Among its most exciting developments is the use of stem cell-based therapies, which have demonstrated considerable potential for repairing and restoring damaged tissues in both medical and dental fields. The craniofacial region, in particular, presents unique challenges due to its complex anatomy and aesthetic-functional importance, making innovative approaches in tissue regeneration especially valuable.

### 1.1. Regeneration with Mesenchymal Stem Cells

Within this context, mesenchymal stem cells (MSCs) have attracted increasing scientific interest for their versatility, accessibility, and regenerative capabilities. Their use in oral and maxillofacial surgery represents a significant advancement, offering new possibilities for the treatment of bone and soft tissue defects resulting from trauma, disease, or surgical intervention [1].

The following sections will provide an overview of the biological characteristics of stem cells, with particular focus on mesenchymal stem cells and their clinical applications in maxillofacial and oral surgery [2].

### 1.2. Stem Cells: Classification and Biological Properties

Stem cells are undifferentiated cells with the unique ability to self-renew and differentiate into specialized cell types [3,4,5,6,7]. Their classification depends on both their potential to differentiate and their source [8,9,10]. Among the different categories, mesenchymal stem cells (MSCs) have drawn significant interest due to their regenerative potential, immunoregulatory properties, and ease of isolation [11,12,13,14]. Stem cells fall into two broad groups: embryonic stem cells and adult stem cells. These cells, derived from the inner cell mass of the blastocyst, are pluripotent, meaning they can differentiate into nearly any cell type in the human body [15,16]. However, their clinical application is hindered by ethical concerns and the risk of tumor formation. In contrast, adult stem cells, which include MSCs, are multipotent and primarily function in tissue repair and regeneration. MSCs have attracted significant research interest because of their therapeutic value and the ease of isolating them from multiple tissue sources [17,18,19,20,21].

### 1.3. Mesenchymal Stem Cells (MSCs)

MSCs are a population of multipotent stem cells capable of differentiating into osteogenic, chondrogenic, adipogenic, and myogenic lineages [22,23,24,25,26,27,28,29,30,31]. They can be harvested from multiple sources, including bone marrow, adipose tissue, and dental pulp [32,33,34,35,36]. Their low immunogenic profile, resulting from the lack of MHC class II expression, makes them well suited for use in allogeneic transplants [37,38,39,40,41,42]. Furthermore, MSCs release bioactive molecules such as cytokines and growth factors that support tissue repair and regeneration [43,44,45,46].

A key feature of MSCs is their immunomodulatory capacity. They interact with the immune system through direct cell-to-cell contact and the secretion of anti-inflammatory factors, including transforming growth factor-beta, interleukin-10, and prostaglandin E2. These properties make MSCs valuable in treating inflammatory and autoimmune disorders, as well as in enhancing healing in maxillofacial procedures where immune responses significantly impact tissue repair [47,48,49,50,51,52].

Mesenchymal stem cells derived from human periapical cysts (hPCy-MSCs) represent a promising resource for regenerative medicine [53]. First isolated in 2013, these cells are located in the inner layer of the cyst wall and exhibit similar characteristics to healthy MSCs, with a high potential for differentiation into osteoblasts, adipocytes, and even dopaminergic neurons [54]. They also possess significant immunomodulatory properties, capable of reducing local inflammation, limiting tissue damage, and promoting bone regeneration. Recent studies suggest that hPCyMSCs may contribute to periapical tissue repair due to their proangiogenic capacity [41]. These cells show affinity for synthetic scaffolds (such as PLGA and chitosan), making them suitable for tissue engineering applications [55]. Furthermore, the inflammatory environment appears to stimulate their activity, suggesting that pathological tissue may not be an obstacle but rather a potential regenerative source.

### 1.4. Bone Marrow-Derived Stem Cells (BMSCs)

*BMSCs*, a well-researched type of MSCs, are obtained from bone marrow aspirates and are recognized for their capacity to differentiate into osteogenic and chondrogenic lineages [56,57,58]. Their role is particularly relevant in maxillofacial surgery, where they contribute to bone regeneration in cases of trauma, congenital defects, or tumor resection. Despite their regenerative potential, the clinical use of BMSCs faces certain obstacles. Bone marrow aspiration is an invasive procedure, and the quantity and quality of BMSCs decline with age. Additionally, BMSCs require in vitro expansion before clinical application, raising concerns regarding cellular senescence and genetic stability.

### 1.5. Adipose-Derived Stem Cells (ADSCs)

ADSCs offer a promising alternative to BMSCs due to their ease of extraction via minimally invasive liposuction procedures and their high proliferative capacity [59,60,61,62]. These cells demonstrate similar multipotent characteristics to BMSCs and are especially beneficial for maxillofacial tissue engineering because of their potential to form bone and cartilage [63,64,65,66,67]. ADSCs are abundant and readily accessible, making them a more practical option for regenerative medicine [68,69,70]. They also play a crucial role in vascularization and wound healing by secreting angiogenic factors such as vascular endothelial growth factor, which stimulates blood vessel formation [71,72]. Their ability to regulate inflammation further enhances their application in maxillofacial surgery, where soft tissue regeneration and healing are critical [73].

### 1.6. Stem Cell-Based Approaches in Maxillofacial and Oral Surgery

The integration of stem cells into maxillofacial and oral surgery has represented one of the major innovations in the field of regenerative medicine, offering new opportunities for the repair and reconstruction of hard and soft tissues [74,75,76,77].

MSCs are particularly well suited for addressing various craniofacial disorders due to their biological traits, notably their capacity to differentiate into bone, cartilage, and fat cell lineages [78,79,80,81,82,83,84,85]. For bone regeneration purposes, stem cells are often integrated with biomaterials and biocompatible scaffolds to enhance bone formation in areas compromised by trauma, inflammation, or major surgical resections. This regenerative strategy overcomes some of the limitations of traditional bone grafts, such as donor site morbidity and the limited availability of autologous tissue, offering a biologically active and customizable alternative [86,87,88,89,90].

In oral surgery, MSCs are employed in alveolar bone regeneration, management of peri-implant bone defects, and guided tissue regeneration [91,92]. Their combination with resorbable membranes and osteoinductive factors allows for more efficient and predictable bone tissue regeneration, improving the conditions for implant placement and reducing healing times [93,94,95,96,97].

Stem cells, especially those sourced from adipose tissue, have significantly advanced soft tissue regeneration. Their ability to promote blood vessel formation and regulate inflammation plays a crucial role in healing delicate areas like the oral mucosa and gingiva. These characteristics also make them ideal for use in facial reconstructive and aesthetic procedures [98,99,100,101].

Another promising application is in the treatment of temporomandibular joint (TMJ) disorders, where MSCs are being explored for their ability to modulate inflammation, regenerate cartilage tissue, and improve joint function [102,103,104]. Although this application is still under development, the therapeutic potential is promising and could lead to minimally invasive biological treatments for degenerative conditions [105,106,107,108].

Finally, dental pulp stem cells (DPSCs) represent a valuable resource for dental tissue regeneration, thanks to their neural origin and their capacity to differentiate into odontoblasts and other relevant cell types for dentin and pulp repair. Their application is expanding in both endodontic tissue regeneration and the restoration of damaged dental structures, offering promising prospects for maintaining tooth vitality. In summary, the use of stem cells in maxillofacial and oral surgery stands out as a versatile and biologically advanced therapeutic strategy, capable of addressing complex clinical needs through the integration of biotechnology, tissue engineering, and regenerative medicine [109,110].

The aim of this systematic review is to critically analyze the use of MSCs, particularly those derived from bone marrow, ADSCs, and dental pulp, in maxillofacial and oral surgery. The review seeks to evaluate the effectiveness and clinical applications of these cells in the regeneration of hard and soft tissues, identifying current preclinical and clinical evidence, therapeutic potential, integration techniques with biomaterials and scaffolds, as well as the main challenges and future perspectives in the field of regenerative medicine.

## 2. Materials and Methods

### 2.1. Protocol and Registration

The systematic review protocol was registered in PROSPERO with the ID 1033255. This review adhered to the guidelines established by the Preferred Reporting Items for Systematic Reviews and Meta-Analyses (PRISMA).

### 2.2. Search Processing

Studies concerning the use of stem cells in maxillofacial and oral surgery were identified by searching PubMed, Scopus, and Web of Science 1 January 2015 and 1 April 2025. The search strategy utilized the following terms: (“stem cells” OR “mesenchymal stem cells” OR “bone marrow-derived stem cells” OR “adipose-derived stem cells”) AND (“maxillofacial” OR “craniofacial” OR “oral surgery”) (Table 1).

### 2.3. Inclusion Criteria

The criteria for including studies were as follows:

1. Focus on stem cell applications in maxillofacial and oral surgery;
2. Types of studies: randomized controlled trials, retrospective research, case–control studies, case series, and prospective studies;
3. Studies published in English;
4. Full-text availability.

Articles that did not fulfill these requirements were excluded from further analysis.

The PICOS criteria were used to conduct the review:

- Participants: Human patients receiving MSC-based regenerative therapies specifically within the field of oral and maxillofacial surgery;
- Interventions: Application of MSCs derived from various sources (e.g., bone marrow, dental pulp, adipose tissue);
- Comparison: conventional regenerative approaches or no treatment controls, when applicable;
- Outcomes: Quantitative and qualitative evaluation of regenerative outcomes, including bone/soft tissue formation, clinical integration, and safety/adverse events;
- Study Design: Prospective and retrospective clinical studies, including RCTs and controlled case series.

### 2.4. Exclusion Criteria

The exclusion criteria were the following:

1. Animal studies;
2. Studies on unrelated topics;
3. Review articles, letters, or commentaries;
4. Studies published in languages other than English.

### 2.5. Data Processing

Three reviewers (M.G., I.P., and R.M.) independently performed database searches and assessed the quality of the retrieved articles. The Zotero version 6.0.15 (Corporation for Digital Scholarship, Vienna, VA, USA) was used to download the selected articles. In cases of disagreement between the reviewers, a senior reviewer (F.I.) was consulted for clarification.

### 2.6. Quality Assessment

The quality of the selected studies was evaluated using the ROBINS tool. The three reviewers (M.G., I.P., and R.M.) assessed the potential bias in the following domains:

- Confounding bias;
- Bias related to exposure measurement;
- Bias in participant selection;
- Bias from post-exposure interventions;
- Bias resulting from missing data;
- Bias from outcome measurement;
- Bias in reporting the results.

## 3. Results

### 3.1. Study Selection and Methodological Features

The electronic database search returned a total of 13,989 publications (PubMed N = 4836, Scopus N = 2516, and Web of Science N = 6637). After removing duplicates (N = 4576), 9413 records were available for screening. These records were assessed based on their titles and abstracts, resulting in the evaluation of 9413 studies. Of these, 8216 studies did not meet the inclusion criteria (8030 off-topic, 171 reviews, and 15 animal studies), leaving 1197 records for further review.

**Figure 1:**
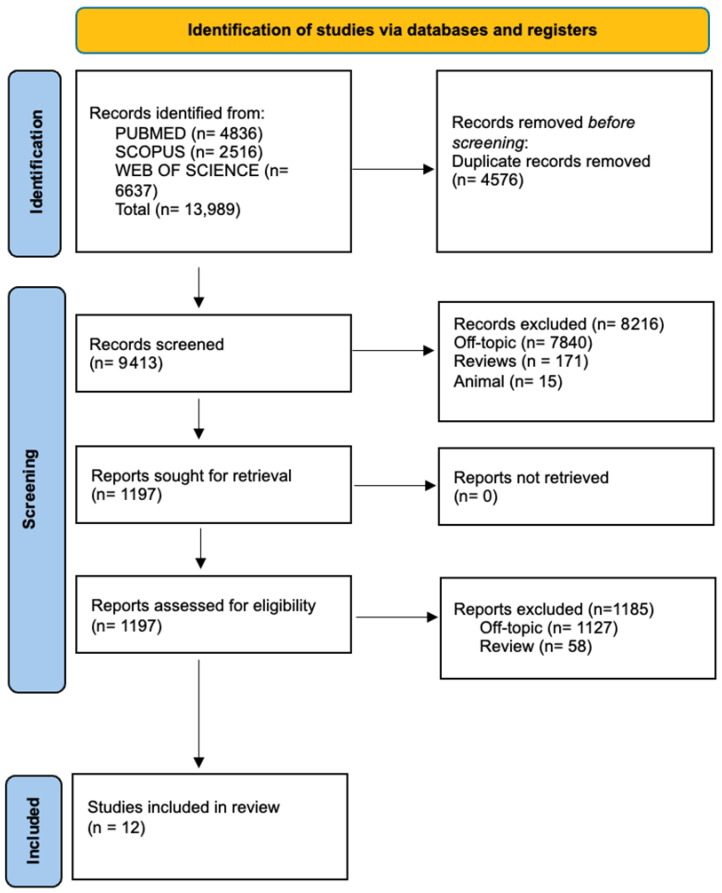
Literature search following the Preferred Reporting Items for Systematic Reviews and Meta-Analyses (PRISMA) flow diagram and database search indicators.

Following full-text screening, 1185 studies were excluded for the following reasons: 1127 were off-topic and 58 were reviews. Ultimately, 12 studies were included in the final review. The selection process is depicted in Figure 1, and a summary of the selected records is provided in Table 2.

### 3.2. Quality Assessment and Risk of Bias

Regarding the bias due to confounding, most studies show some concerns, with 10 studies falling into this category and only 2 studies assessed as low risk. The bias arising from measurement of the exposure is generally low, with eight studies rated as low risk and four showing some concerns. Many studies have a low risk of bias in the selection of participants, with six studies categorized as low risk and six presenting some concerns. Bias due to post-exposure interventions is mostly low, with eight studies showing low risk, three with some concerns, and one with high risk, although some heterogeneity is observed across studies. The bias due to missing data presents mostly some concerns, with seven studies in this category and five with a low risk of bias. Bias arising from the measurement of the outcome is predominantly low, with eight studies classified as low risk, three with some concerns, and one with a high risk. Finally, the bias in the selection of the reported results shows some concerns in most studies, with seven studies in this category and five rated as low risk.

**Figure 2:**
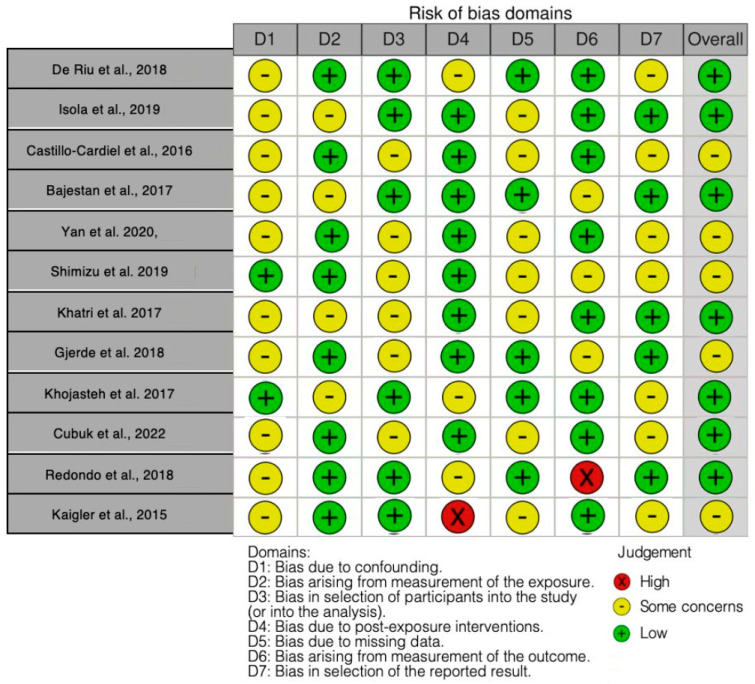
Bias assessment. The figure highlights the proportion of studies categorized as having low, high, or unclear risk of bias in each demain, providing a visual summary of the overall methological quality of the included studies. De Riu et al., 2018 [111]; Isola et al., 2019 [112]; Castillo-Cardiel et al. (2016) [113]; Bajestan et al. (2017) [114]; Yan et al. (2020) [115]; Shimizu et al. (2019) [116]; Khatri et al., 2017 [117]; Gjerde et al., 2018 [118]; Khojasteh et al. (2017) [119]; Cubuk et al. (2022) [120]; Redondo et al., 2018 [121]; Kaigler et al., 2015 [122].

The final results indicate that out of the analyzed studies, seven have a low risk of bias and five present some concerns (Figure 2).

## 4. Discussion

Stem cell therapies have garnered considerable interest in oral and maxillofacial surgery owing to their regenerative capabilities, especially for bone regeneration and tissue repair. MSCs, especially those derived from bone marrow and adipose tissue, are commonly utilized in this field for their unique biological properties. These therapies have been investigated in various clinical settings, with promising, yet mixed results.

De Riu et al. (2018) explored the use of bone marrow nucleated cells (BMNc) for the treatment of TMJ disorders. The study revealed that BMNc injections were more effective than hyaluronic acid (HA) in providing pain relief, improving chewing efficiency, and enhancing maximum interincisal opening over a 12-month period. However, no significant changes were observed in joint noises or cartilage regeneration as seen in MRI scans, suggesting that while BMNc therapy shows promise, further research is needed to establish its long-term effectiveness and safety in treating degenerative TMJs [111].

Similarly, Isola et al. (2019) examined the impact of periodontitis and tooth loss on endothelial progenitor cell (EPC) levels, specifically CD133+/KDR+ cells. The study found that patients with periodontitis had significantly lower EPC levels compared to healthy controls, which correlated with increased severity of periodontal disease. This finding underscores the potential role of EPCs in vascular repair and suggests that periodontal health might influence regenerative processes in other tissues, including bone. Although the study does not directly focus on stem cell therapy, it highlights the importance of maintaining periodontal health for optimal regenerative outcomes [112].

On the other hand, Castillo-Cardiel et al. (2016) assessed the use of autologous mesenchymal stem cells (AMSCs) for mandibular fracture healing. Their randomized controlled trial demonstrated that AMSCs derived from adipose tissue could significantly improve bone regeneration in mandibular fractures. At 12 weeks, the AMSCs group showed a 36.48% higher ossification rate compared to the control group, highlighting the potential of AMSCs in enhancing bone quality and reducing healing time. However, the study did not compare AMSCs with other stem cell sources, and further studies are needed to confirm the superiority of AMSCs in bone regeneration [113].

Bajestan et al. (2017) evaluated stem cell therapy for the regeneration of large alveolar defects in adults with cleft palate or craniofacial trauma. The results indicated that while stem cell therapy showed safety and promise, it was less effective than conventional autogenous bone grafts in achieving significant bone gain. The stem cell therapy group gained 1.5 mm of bone width, compared to 3.3 mm in the control group. Despite the limited bone regeneration in the stem cell group, the approach was deemed safe and showed potential as an alternative treatment for alveolar defects. The study highlighted that stem cell therapy requires further optimization before it can compete with established grafting methods [114].

There is growing interest in the use of MSCs, particularly those derived from dental pulp and bone marrow, for craniofacial bone regeneration. A central focus across multiple studies has been the impact of cryopreservation on the viability and regenerative capacity of DPSCs. Cryopreservation, especially with the use of dimethyl sulfoxide (DMSO), was shown to delay initial cellular outgrowth and attachment in vitro. However, DPSCs preserved with 5% DMSO demonstrated better performance in terms of outgrowth time compared to those preserved with 10% DMSO, while maintaining biological activity and differentiation potential into osteogenic and adipogenic lineages [115]. These findings highlight the feasibility of utilizing cryopreserved dental pulp tissue as a cell source for regenerative applications, provided that optimal preservation conditions—particularly lower DMSO concentrations—are employed to minimize cytotoxic effects and preserve multipotency [117].

In parallel, clinical trials investigating the therapeutic application of MSCs for bone regeneration have reported promising, albeit varied, outcomes. The TEOM study, a well-structured randomized controlled trial, explored the safety and efficacy of autologous BMSCs in patients with extensive maxillomandibular bone defects. Preliminary data suggest that BMSCs can enhance bone regeneration, with secondary outcomes such as implant stability and histological quality indicating the potential for clinical translation. However, definitive conclusions await long-term data from the ongoing 24-month follow-up [116].

Similarly, another clinical trial demonstrated that autologous BMSCs combined with biphasic calcium phosphate scaffolds effectively increased alveolar ridge dimensions in the posterior mandible. The study observed a significant mean horizontal bone gain of over 4 mm, along with new bone formation confirmed by histological and micro-CT analyses. Importantly, all patients achieved sufficient bone volume for implant placement without severe adverse effects, underscoring the therapeutic promise of MSC-based interventions in challenging anatomical contexts [118].

A novel approach involving buccal fat pad-derived MSCs (BFSCs) also yielded favorable outcomes in alveolar cleft reconstruction. When combined with natural bone mineral matrices, BFSCs significantly enhanced bone regeneration, particularly in the group using anterior iliac crest spongy bone. This intraoral MSC source, along with the demonstrated high expression of MSC markers and differentiation potential, suggests that BFSCs may serve as an accessible and effective cell type for craniofacial applications. Nonetheless, validation through studies with larger cohorts and additional growth factor supplementation is necessary to refine these findings [119].

The study introduces a novel, minimally invasive procedure for isolating multipotent progenitor cells (MPCs) from small tonsillar biopsy samples, typically weighing less than 1 g. The procedure avoids the complications associated with major surgical interventions and yields a high number of viable, highly proliferative MPCs that can be massively expanded in culture. These cells express key mesenchymal progenitor markers and demonstrate robust differentiation potential into osteogenic, adipogenic, and chondrogenic lineages, comparable to bone marrow-derived MPCs. Furthermore, T-MPCs can be cultured in xeno-free conditions, making them suitable for regenerative medicine and cell therapy. The ability to harvest MPCs from tonsillar biopsies offers a promising, low-risk alternative to current methods relying on discarded tissue from major surgeries, presenting significant advantages for both autologous and allogeneic cell therapies [117].

Collectively, the available data reinforce the regenerative potential of MSCs derived from both dental pulp and bone marrow, especially when paired with optimized cryopreservation and scaffold strategies. Although variations in clinical outcomes remain, these approaches offer promising alternatives to traditional grafting methods. Future research should prioritize standardization of cell processing, scaffold design, and delivery mechanisms to ensure consistent clinical efficacy and broaden the applicability of MSC-based therapies in oral and maxillofacial bone regeneration.

Cubuk et al. evaluated the clinical and radiographic efficacy of DPSCs seeded onto leukocyte- and platelet-rich fibrin (L-PRF), compared to L-PRF alone, for socket preservation following extraction of impacted mandibular third molars. In a split-mouth randomized controlled trial involving 13 patients, both groups showed significant improvements in probing pocket depth, clinical attachment levels, and vertical bone loss after six months. However, the addition of DPSCs did not yield significantly better outcomes compared to L-PRF alone. These findings suggest that while L-PRF is effective for socket preservation, the adjunctive use of mechanically disaggregated DPSCs may not provide substantial additional benefits in terms of periodontal parameters of adjacent second molars [120]. Other studies underscore the regenerative potential of MSCs when combined with appropriate scaffolds. Redondo et al. (2018) employed AMSCs seeded on cross-linked serum scaffolds (BioMax, Vicenza, Italy) to treat cystic lesions of the maxilla. The constructs underwent osteogenic differentiation prior to implantation. After seven months, the treated sites demonstrated a significant increase in bone density (post/pre ratio: 2.52 ± 0.45, *p* < 0.005), with no evidence of adverse reactions or inflammation. The BioMax scaffold exhibited excellent biocompatibility, osteoconductivity, and support for MSC proliferation. This strategy represents a promising approach for maxillofacial bone regeneration and may be further optimized by incorporating allogeneic cells to improve clinical feasibility [121]. Kaigler et al. also demonstrated the efficacy of stem cell therapy in oral reconstruction. In their study, autologous bone marrow-derived cells enriched with CD90+ stem cells and CD14+ monocytes were compared with β-tricalcium phosphate scaffold alone for maxillary sinus floor augmentation. While both interventions achieved comparable total bone volumes, the cell-enriched therapy resulted in significantly greater bone density and quality, particularly in cases with severe bone loss (>50% height reduction). A positive correlation was observed between the percentage of CD90+ cells and bone volume fraction (r = 0.56; *p* = 0.05), suggesting improved regenerative outcomes with higher stem cell concentrations. All implants achieved successful osseointegration and functional loading without adverse events, supporting the safety and efficacy of stem cell-enhanced bone regeneration for complex maxillofacial defects. Furthermore, AMSCs have shown promise in the treatment of mandibular fractures [122]. In summary, current evidence supports the use of MSCs, either in combination with biocompatible scaffolds or as stand-alone cellular therapies, as a promising strategy for maxillofacial bone regeneration. However, further large-scale randomized controlled trials are needed to validate these findings and to establish standardized protocols for their clinical application.

## 5. Limitations

Despite the promising potential of MSC-based therapies in oral and maxillofacial surgery, several limitations remain. Many studies feature small sample sizes, short follow-up periods, and heterogeneity in cell sources, preparation protocols, and scaffold materials, making direct comparisons challenging. Variability in clinical outcomes may be influenced by defect characteristics, vascularization, and host–cell interactions. Moreover, MSC-based approaches have sometimes demonstrated inferior results compared to conventional grafting techniques, particularly in complex defects such as alveolar clefts. The lack of standardized protocols for cell isolation, dosing, scaffold design, and delivery further hinders clinical translation. Lastly, the long-term safety, functionality, and stability of regenerated tissues are still insufficiently documented.

## 6. Conclusions

MSC-based therapies represent a promising frontier in oral and maxillofacial regenerative medicine, offering potential advantages over traditional grafting techniques, particularly in enhancing bone regeneration, reducing healing times, and minimizing donor site morbidity. The use of MSCs from various sources—such as bone marrow, adipose tissue, dental pulp, and buccal fat pad—combined with biocompatible scaffolds and optimized cryopreservation methods, has demonstrated encouraging results across a range of clinical applications. However, the overall heterogeneity in study designs, cell processing techniques, and clinical endpoints underscores the need for greater standardization. While safety profiles are generally favorable, further research is essential to determine long-term efficacy, establish best practices, and identify the most suitable cell–scaffold combinations for different defect types. Continued progress in this field relies on robust, well-controlled clinical trials and interdisciplinary collaboration to translate regenerative strategies into predictable and widely accessible clinical treatments.

## Figures and Tables

**Table 1:** Full search strings for each database.

Database	Search String
PubMed	(“stem cells” OR “mesenchymal stem cells” OR “bone marrow-derived stem cells” OR “adipose-derived stem cells”) AND (“maxillofacial” OR “craniofacial” OR “oral surgery”)
Scopus	(“stem cells” OR “mesenchymal stem cells” OR “bone marrow-derived stem cells” OR “adipose-derived stem cells”) AND (“maxillofacial” OR “craniofacial” OR “oral surgery”)
Web of Science	(“stem cells” OR “mesenchymal stem cells” OR “bone marrow-derived stem cells” OR “adipose-derived stem cells”) AND (“maxillofacial” OR “craniofacial” OR “oral surgery”)

**Table 2:** Descriptive summary of item selection.

Author (Year)	Study Design	Number of Patients	Average Age and Gender	Stem Cells Used	Outcomes
De Riu et al., 2018 [111]	RCT	30	Not Specified	BMNc	Pain relief, better chewing, increased mouth opening at 6–12 months.
Isola et al., 2019 [112]	Observational study	167	Not specified	Endothelial Progenitor Cells	Lower EPCs linked to worse periodontal disease.
Castillo-Cardiel et al. (2016) [113]	Randomized clinical trial	20 (10 per group)	31.2 ± 6.3 years (study group), 29.7 ± 7.2 years (control group), all male	Autologous MSCs (AMSCs)	Improved bone quality; 36.48% higher ossification at 12 weeks.
Bajestan et al. (2017) [114]	Randomized controlled clinical trial	18 (10 with trauma, 8 with cleft palate)	Not specified	Bone marrow-derived MSCs	Less bone gain; 5/10 implant success in stem cell group.
Yan et al. (2020) [115]	Experimental laboratory study	10 healthy children (aged 10–15 years)	Not specified	DPSCs isolated from dental pulp tissue of extracted third molars.	Cryopreservation slightly delayed cell outgrowth, no major functional impact.
Shimizu et al. (2019) [116]	Randomized controlled trial	29 patients	Patients aged 20+ years	BM-MSCs derived from iliac crest bone marrow	Successful bone regeneration (CT ≥ 400, height > 10 mm).
Khatri et al., 2017 [117]	Sperimental study	10	9 Female and 5 male	T-MPCs (tonsil-derived mesenchymal progenitor cells)	Tonsils as viable stem cell source for research/clinical use.
Gjerde et al., 2018 [118]	Clinical trial	11 patients	52–79 years	Bone marrow-derived MSCs	New bone formation without adverse effects.
Khojasteh et al. (2017) [119]	Prospective randomized clinical trial.	Ten patients	Four adult patients (20–29 years old) and six pediatric patients (8–14 years old) 3 female	MSCs derived from the buccal fat pad (BFP)	Enhanced regeneration, reduced resorption with scaffold.
Cubuk et al. (2022) [120]	Split-mouth RCT	13 patients	23.6 ± 4.4 years; 7F, 6M	DPSCs	Both groups improved; no difference with or without DPSCs.
Redondo et al., 2018 [121]	RCT	9 patients	38 ± 5 years (7F, 2M)	Autologous bone-derived mesenchymal stem cells	Bone density increased, no rejection, effective regeneration.
Kaigler et al., 2015 [122]	RCT	26 patiens	Not specified	Autologous bone marrow-derived cells enriched with CD90+ stem cells and CD14+ monocytes	Higher bone volume; successful implants; no adverse events.

## Abbreviations

ADSCs	Adipose-derived stem cells
AMSCs	Autologous mesenchymal stem cells
BFSCs	Buccal fat pad-derived MSCs
BMNc	Bone marrow nucleated cells
BMSCs	Bone marrow stem cells
CD133+/KDR+	Cell surface markers for endothelial progenitor cells (CD133 and KDR)
DMSO	Dimethyl sulfoxide
EPC	Endothelial progenitor cell
HA	Hyaluronic acid
L-PRF	Platelet-rich fibrin
MPCs	Multopotent progenitor cells
MSCs	Mesenchymal stem cell
TMJ	Temporomandibular joint
